# Case Report: Cutaneous *Mycobacterium chelonae* infection in a kidney transplant recipient with long-term immunosuppression and eculizumab therapy

**DOI:** 10.3389/fmed.2025.1728249

**Published:** 2026-01-12

**Authors:** Diana Sukackiene, Deimante Satkauskaite, Ugne Sleivyte, Arturas Vinikovas, Gintare Ulianskaite, Justinas Pamedys, Tadas Raudonis, Marius Miglinas

**Affiliations:** 1 Institute of Clinical Medicine, Faculty of Medicine, Vilnius University, Vilnius, Lithuania; 2 National Centre of Pathology Affiliated to Vilnius University Hospital Santaros Klinikos, Vilnius, Lithuania

**Keywords:** eculizumab, immunossuppression, kidney transplantation, mycobacteria chelonae, nontubercolous mycobacteria

## Abstract

*Mycobacterium chelonae* is a rapidly growing non-tuberculous mycobacterium (NTM) that causes skin and soft tissue infections, particularly in immunocompromised patients. We present the case of a 51-year-old woman receiving eculizumab therapy after kidney transplantation, who developed painful nodules and ulcers on the lower leg. Standard laboratory and imaging findings were unremarkable. Initial cultures were negative, but biopsy and subsequent wound cultures identified *M. chelonae*. Repeat testing confirmed the pathogen. The patient was successfully treated with a 12-month course of combination antibiotics, including clarithromycin, linezolid, and doxycycline, along with intralesional gentamicin. A follow-up biopsy showed dermal fibrosis without microorganisms. This case highlights the diagnostic challenges of *M. chelonae* in transplant recipients and emphasizes the importance of molecular diagnostics and tailored therapy. To the best of our knowledge, this is the first reported case of *M. chelonae* infection in a kidney transplant recipient receiving eculizumab as part of her post-transplant management.

## Case presentation

A 51-year-old woman presented with nodules and ulcers on her right lower extremity, which developed 1 month following her fourth kidney transplant. Her medical history was significant for thrombotic microangiopathy during her first pregnancy, resulting in the failure of prior transplants and the subsequent diagnosis of a heterozygous factor H (C448Y) mutation. She is currently undergoing eculizumab therapy for atypical hemolytic uremic syndrome. In addition, the patient had been on long-term maintenance immunosuppression, including systemic corticosteroids (methylprednisolone), tacrolimus, and mycophenolate mofetil.

Physical examination revealed violaceous-cyanotic nodules (approximately 1 cm in diameter) on the right lower leg, concentrated around the ankles and extending proximally, accompanied by localized swelling. Several nodules were ulcerated, with small superficial ulcers exuding yellowish discharge. These painful lesions arose spontaneously without trauma. Proximally, new erythematous, warm nodules suggested ongoing infectious progression, with overall worsening despite initial topical therapy.

General blood tests and biochemical analyses were within normal limits. Histopathological examination of a punch biopsy revealed findings consistent with erythema nodosum. No cytomegalovirus (CMV) or SV40 cells were identified. Gentamicin cream was prescribed for the ulcers, to be applied once daily for 14 days, and betamethasone ointment was prescribed for the surrounding skin, to be applied once daily for 30 days. Consultations with a dentist, otolaryngologist, and gynecologist did not identify any sources of infection. No inflammatory changes, tuberculosis mycobacteria, or non-tuberculous mycobacteria were detected in the wounds. Chest computed tomography (CT) and chest X-ray revealed no significant pathological changes in the lungs.

**Figure 1:**
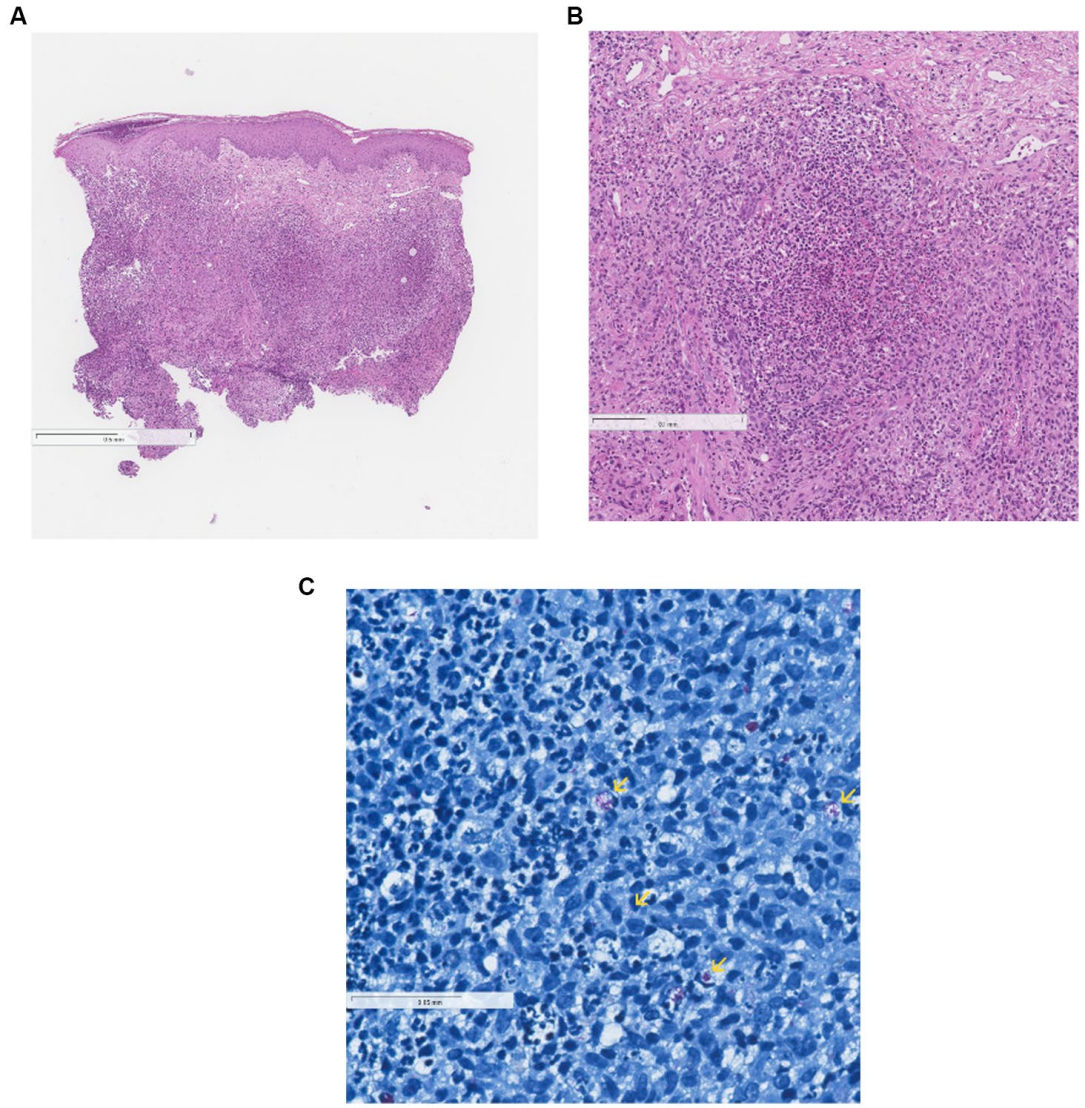
**(A,C)** Histopathological findings consistent with *Mycobacterium chelonae* infection. **(A)** Skin biopsy sample with suppurative, poorly formed granulomatous inflammation in the dermis (hematoxylin–eosin stain, 40 × magnification). **(B)** Suppurative, poorly formed granulomatous inflammation in the dermis (hematoxylin–eosin stain, 100 × magnification). **(C)** Rod-shaped acid-fast bacilli (arrows) dispersed among areas of suppurative inflammation (Ziehl–Neelsen stain, 400 × magnification).

Two months following the initial presentation, wound culture analysis identified a significant growth of *Mycobacterium chelonae*. Antimicrobial therapy with ciprofloxacin was initiated. While the skin lesions showed gradual improvement, erythematous nodules remained. Three months later, concerns about potential contamination of the initial sample with non-tuberculous mycobacteria prompted a repeat skin biopsy and culture. Histopathological examination confirmed findings consistent with *M. chelonae* infection, including chronic active inflammatory infiltrates, abscess formation, and focal tissue lesions (Figure 1). Subsequent microbiological analyses included two additional cultures, with the second culture yielding a definitive confirmation of *M. chelonae* infection with antibiotic sensitivity. A molecular diagnostic test was conducted to definitively confirm the presence of *M. chelonae.*

**Figure 2:**
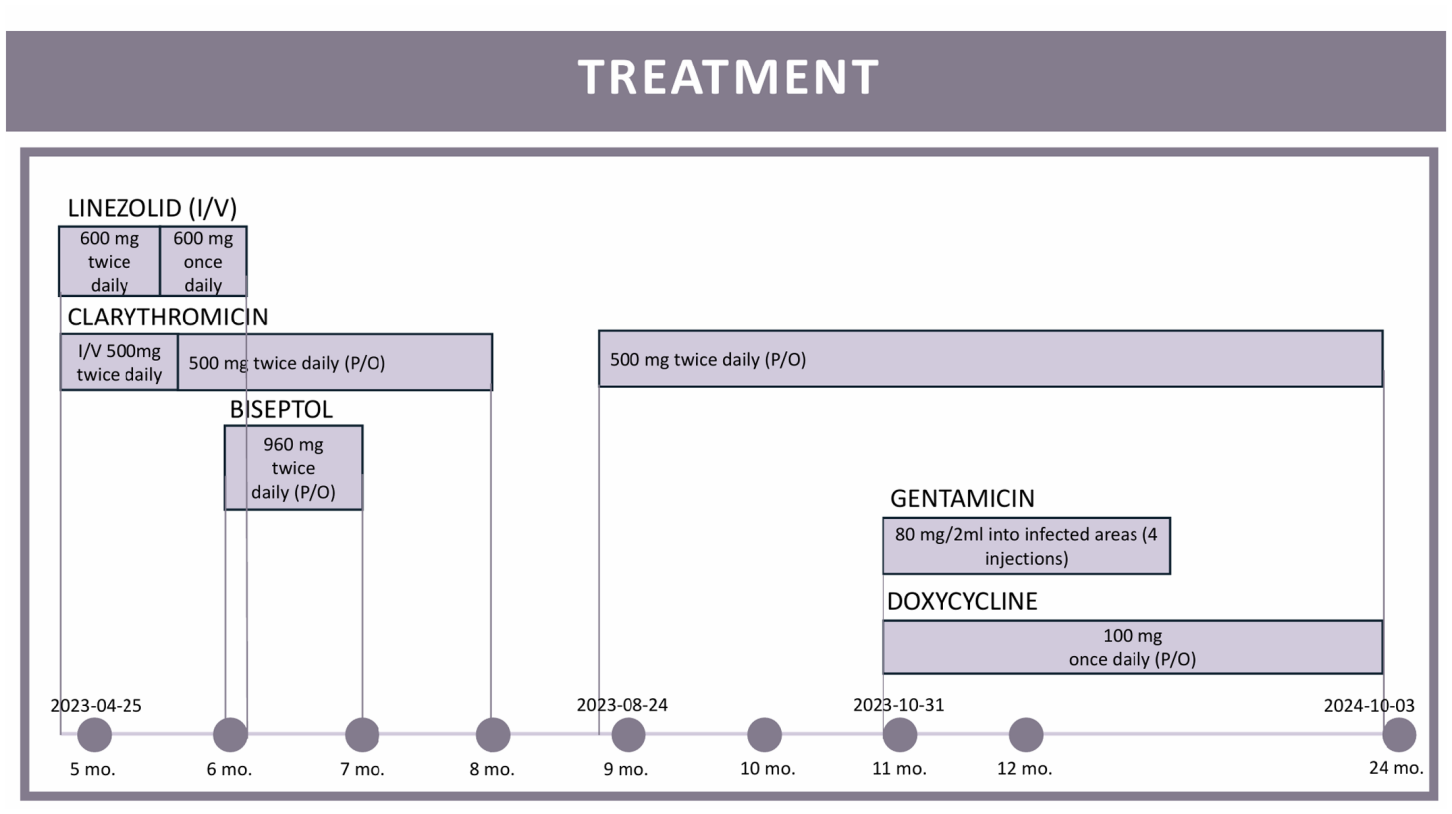
Timeline of administered antibiotic therapy for *Mycobacterium chelonae* infection.

**Figure 3:**
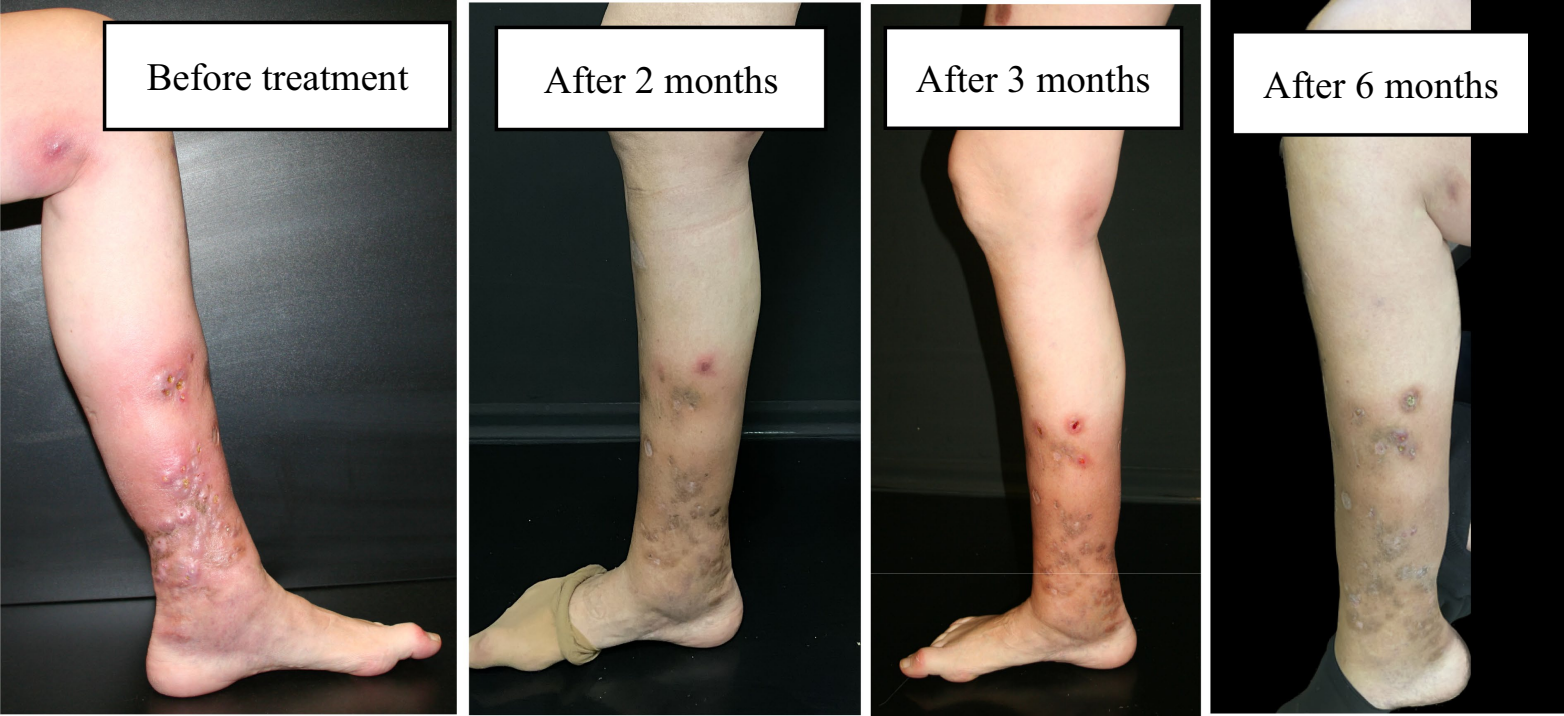
Dynamics of skin lesions during treatment.

**Figure 4:**
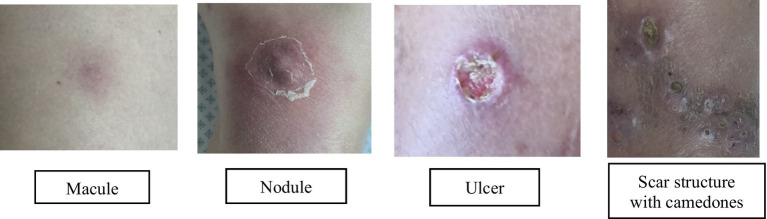
Dynamics of skin lesion.

Progressive lesion exacerbation and clinical decline prompted inpatient admission. We initiated intravenous linezolid (600 mg twice daily) and clarithromycin (500 mg twice daily), transitioning to oral clarithromycin (500 mg BID for 4 months) with adjunctive doxycycline (100 mg daily) due to suboptimal response (Figure 2: treatment timeline and lesion evolution). Four intralesional gentamicin injections (80 mg each) yielded notable improvement. Repeat cultures were sterile throughout. After 12 months of combination therapy, symptoms resolved: swelling and pain abated, nodules regressed, and only post-inflammatory hyperpigmentation remained (Figures 3,4). Palpable subcutaneous nodules likely represented scar tissue. A follow-up biopsy showed granulomatous changes with dermal fibrosis but no microorganisms, prompting treatment discontinuation given clinical resolution and negative microbiology.

## Discussion

*Mycobacterium chelonae* is one of the most pathogenic, rapidly growing non-tuberculous mycobacteria (NTM), classified as group IV under Runyon’s classification. An increase in infections caused by non-tuberculous mycobacteria, including *Mycobacterium chelonae*, was observed in data from a study conducted between 2000 and 2009 (1). While colonies of most rapidly growing NTMs form within 7 days at an optimal temperature of 30–32 °C, *M. chelonae* requires a longer incubation period, averaging 15 days. It is a non-motile, non-spore-forming, Gram-positive, acid-fast bacterium. Although *M. chelonae* infections may remain asymptomatic, immunosuppressed patients often present with localized or disseminated skin infections, post-procedural infections, or catheter-associated infections (2).

Cutaneous infections caused by *M. chelonae* are well recognized in solid-organ transplant recipients, with a reported incidence of NTM disease after kidney transplantation of 0.16–0.55% (3–5). The dominant risk factor is prolonged T-cell and macrophage dysfunction from calcineurin inhibitors, antimetabolites, and chronic corticosteroid exposure—a classic high-risk scenario in patients with multiple prior transplants. With over 20 years of continuous immunosuppression across four grafts, our patient was at the extreme end of risk for rapidly growing mycobacterial infections.

To the best of our knowledge, this is the first case of *M. chelonae* infection in a patient receiving eculizumab. Terminal complement inhibition dramatically increases susceptibility to encapsulated bacteria (especially Neisseria spp.) by blocking the membrane attack complex and weakening C5a-driven neutrophil recruitment. Whether this worsens control of NTM is unclear—these organisms are primarily controlled by interferon-gamma (IFN-*γ*)-activated macrophages, a pathway eculizumab leaves intact (6, 7).

Limited *in vitro* data suggest that C5b-9 can damage some mycobacterial species, and C5a enhances monocyte recruitment; thus, in the setting of already profound conventional immunosuppression, terminal complement inhibition might theoretically exert an additive effect. However, large pharmacovigilance cohorts of eculizumab-treated patients (atypical hemolytic uremic syndrome [aHUS] and paroxysmal nocturnal hemoglobinuria) and the recent era of other complement inhibitors (ravulizumab and pegcetacoplan) have not reported an increased incidence of NTM disease (8).

Therefore, while we cannot exclude a contributory role, the cumulative burden of decades of transplant immunosuppression remains the primary predisposing factor in this case, as there were no evident trauma sites or invasive procedures at the site of infection. Similar cases have been reported in the literature (3, 6). Diagnosis of *M. chelonae* is frequently delayed because routine bacterial cultures do not support growth and early histology may mimic erythema nodosum or vasculitis—as occurred here. Current clinical practice guidelines by the American Thoracic Society (ATS), European Respiratory Society (ERS), European Society of Clinical Microbiology and Infectious Diseases (ESCMID), and Infectious Diseases Society of America (IDSA) emphasize the need for prolonged incubation, acid-fast staining, and molecular confirmation. Treatment requires prolonged (≥ 6–12 months in immunosuppressed patients) combination therapy guided by susceptibility testing; macrolide-based regimens with a second or third agent (linezolid, doxycycline, aminoglycosides, and clofazimine) are standard (9).

## Conclusion

We present the first reported case of cutaneous *M. chelonae* infection in a kidney transplant recipient receiving eculizumab for aHUS. While the patient’s extreme cumulative immunosuppressive burden over four decades was the principal risk factor, concomitant C5 inhibition may theoretically have contributed. The case underscores the critical importance of repeated tissue sampling with dedicated mycobacterial processing in transplant patients with unexplained skin lesions.

## Data Availability

The original contributions presented in the study are included in the article/supplementary material, further inquiries can be directed to the corresponding author/s.
